# Andersen’s utilization model for cataract surgical rate and empirical evidence from economically-developing areas

**DOI:** 10.1186/s12886-021-01858-x

**Published:** 2021-02-26

**Authors:** Senlin Lin, Yingyan Ma, Zhiyuan Hou, Nathan Congdon, Lina Lu, Haidong Zou

**Affiliations:** 1 Shanghai Eye Diseases Prevention & Treatment Center / Shanghai Eye Hospital, No. 380 Kang Ding Road, Shanghai, 200000 China; 2 Shanghai Key Laboratory of Ocular Fundus Diseases, Shanghai General Hospital, Shanghai Engineering Center for Visual Science and Photomedicine, No. 100 Hai Ning Road, Shanghai, 200000 China; 3 Department of Social Medicine, School of Public Health, National Key Laboratory of Health Technology Assessment (National Health and Family Planning Commission), Collaborative Innovation Center of Social Risks Governance in Health, Fudan University, 138 Yi Xue Yuan Road, Shanghai, 200032 China; 4 State Key Laboratory of Ophthalmology, Zhongshan Ophthalmic Center, Sun Yat-sen University, 54 S. Xianlie Road, Guangzhou, 510060 China; 5 Centre for Public Health, Queen’s University Belfast, Belfast, BT12 6BJ UK; 6 Orbis International, 520 8th Ave #12, New York, NY 10018 USA

**Keywords:** Cataract surgical rate, Developing country, Health service utilization

## Abstract

**Background:**

Un-operated cataract is the leading cause of vision loss worldwide, responsible for 33% of visual impairment, and half of global blindness. The study aimed to build a fast evaluation method utilizing Andersen’s utilization framework and identify predictors of cataract surgical rate in sub-Saharan Africa and China.

**Methods:**

The study was a cross-over ecological epidemiology study with a total of 19 countries in sub-Saharan Africa, and 31 provinces in China. Information was extracted from public data and published studies. Linear regression and structural equation modeling with Bootstrap were used to analyze predictors of CSR and their pathways to impact in sub-Saharan Africa and China separately.

**Results:**

Cataract surgical resources in sub-Saharan Africa were linearly correlated with CSR (β = 0.74, 95% CI: 0.09, 0.91), while GDP/P didn’t impact cataract surgical resources (β = 0.29, 95% CI: − 0.12, 0.75). In China, residents’ average ability to pay was confirmed as the mediator between GDP/P and CSR (*p* = 0.32, RMSEA = 0.07; β_CSR-paying_ = 0.77, 95% CI: 0.25, 0.90; β_paying-GDP/*P*_ = 0.89, 95% CI: 0.82, 0.93).

**Conclusions:**

In sub-Saharan Africa, CSR is determined by health care provision. Local economic development may not directly influence CSR. Therefore, international assistance aimed to providing free cataract surgery directly is crucial. In China, CSR is determined principally by health care demand (ability to pay). To increase CSR in underserved areas of China, ability to pay must be enhanced through social insurance, and reduced surgical fees.

## Background

Un-operated cataract is the leading cause of vision loss worldwide, responsible for 33% of visual impairment, and half of global blindness [1]. Fortunately, vision loss due to cataract is reversible with surgery [2]. Therefore, cataract surgery has been recommended by the World Health Organization (WHO) as critical primary eye care service [2], and is one of the most important components of VISION 2020. It has been shown that surgery for cataract increases the economic well-being of affected patients and their families [3], an impact persisting over at least 6 years [4]. Thus, ready access to cataract surgery is important for achieving the Sustainable Development Goals (SDGs) of improved health, and poverty alleviation. The cataract surgical rate (CSR), defined as the number of cataract surgeries performed per year, per million population, is one of the key indicators in WHO reports.

Although the CSR is high in many wealthier countries, it remains low in under-served regions of low and middle income countries (LMICs): 500–2000 in Asia, and Latin America; < 500 in parts of Africa [5]. To remediate this disparity and aid in achieving the SDGs, the specific predictors of CSR must be identified in under-served areas, in order to formulate intervention strategies.

Numerous studies have identified different predictors of CSR, of which Taylor has summarized the three most important [6]: the population age structure, the vision threshold for surgery, and surgical coverage (effectiveness of healthcare service delivery). These three factors have been widely confirmed, with additional factors having been identified in subsequent work, including gender [7], income [8], availability of health insurance [9], and socio-economic development [5].

However, predictors of CSR have not been consistent across studies conducted in different regions of the world. For example, population age distribution was reported to be associated with CSR in Canada [10], but not in various LMICs. In some LMICs, financial hardship is the major barrier to cataract surgical uptake [11]. In view of such variations, a systematic analytical method is needed to assess the specific predictors of CSR in various regions, in order to develop locally-appropriate interventions for improvement.

Andersen’s healthcare utilization model is the most widely-used theoretical framework in health service research (HSR), and has been verified in many different contexts. The model was established by Andersen and Newman in 1973 and has been modified several times since [12, 13]. It provides a systematic analytical framework with which to explore and analyze factors impacting healthcare utilization in a particular setting, and the pathways through which they work. For example, with the application of Andersen’s model in Arizona, different patterns of healthcare service use were distinguished among older Chinese and Korean immigrants, allowing for the planning of specific interventions in the different communities [14]. To the best of our knowledge, no study has used Anderson’s model to analyze the predictors of cataract surgical uptake, reflected by CSR, in various populations.

The aim of the present study is to identify widely-accepted predictors of CSR, build a specific model based on Andersen’s general framework, and apply this to the specific contexts of sub-Saharan Africa and China, in order to identify key factors for intervention in these regions. Sub-Saharan Africa was chosen as typical of low-income regions, and China to represent middle-income countries. In addition, numerous studies in these regions have been performed in recent decades [15–19], providing data to test the reliability of our model.

## Methods

### Model and indicators

According to Andersen’s model [12, 13], healthcare utilization is impacted by both provision of and demand for healthcare, while healthcare demands are further impacted by healthcare needs, predisposing characteristics, and enabling factors. Environment contexts, such as economic development, may impact most of these other factors, and thus impact healthcare utilization indirectly. Indicators must be selected for each pathway in order to build a specific model for CSR (shown in Supplement 1) [6].

#### Healthcare provision

The numbers of ophthalmologists (or general doctors) per 1000 population were used to indicate the workforce provisions in the sample regions since clinical human resources was found to correlate highly with CSR [20]. Material resources were not included in our model due to lack of evidences about the link between clinical materials and CSR.

#### Healthcare needs

Healthcare needs represents the proportion of individuals needing cataract surgery, which is highly correlated with ageing [21]. Hence, the proportion of the population above the age of 60 years was used to reflect the cataract surgery needs.

#### Predisposing characteristics

Gender structure was chosen as the main indicator of predisposing characteristics, as several studies indicate that gender impacts eye care utilization, especially that of cataract surgery [7, 12, 13].

#### Enabling factors

Anderson suggests that ability to pay is the most important enabling factors [12, 13]. Therefore, annual consumption/income per capita and health insurance financing per capita were selected as the main indicators in our model.

#### Environmental contexts

Economic development has been reported to be highly correlated with CSR [5], and is one of the most important environmental contexts according to Andersen [12, 13]. Gross domestic product per capita (GDP/P) was selected as the main indicator in the present study [5].

### Data sources

Information on CSR, and availability of cataract surgeons in sub-Saharan Africa was extracted from a recent Africa regional VISION 2020 planning document [22], while data on GDP/P, average income, and population age and sex distribution were extracted from *World Bank Open Data*.

Information on CSR in China was extracted from the website of the China National Blindness Prevention and Treatment Organization, data on GDP/P, indicators of healthcare provision, and ability to pay were extracted from *The Chinese National Statistics Year Book 2015*, and information on population age, and sex distribution was extracted from *the Sixth National Population Census*.

### Analysis and statistics

The relationships between CSR (main outcome) and healthcare provision, healthcare needs, gender structure, ability to pay, and GDP/P were first tested separately using Pearson’s correlation. Principal component analysis was performed if multicollinearity existed, which suggested some raw indicators were highly correlated.

Among those factors that correlated with CSR, linear regression was performed using the bootstrap method after data standardization (transformation to mean = 0 and standard deviation = 1) to explore the importance of each indicator.

Mediation analysis was then performed by structural equation modeling (SEM) to explore the deeper relationships. According to the Andersen’s model, healthcare provision, healthcare needs, predisposing characteristics, and enabling factors might be the mediators between economic development and CSR. Therefore, if CSR is correlated with GDP/P, the pathway by which GDP/P causes a change in CSR should be further analyzed.

In addition, because the sample sizes were small (< 50), the Bootstrap method was applied to provide accurate, and robust estimators. In the SEM, the Bollen-Stiner Bootstrap was used to evaluate the goodness-of-fit, and Bootstrap Maximum Likelihood was used to estimate the 95% Confidence Intervals (CIs) of coefficient estimators. All of data cleaning, and analysis was performed with SPSS (IBM SPSS Statistics for Windows, Version 22.0. Armonk, NY: IBM Corp).

### Role of the funding source

The funders of the study had no role in study design; data collection, analysis, or interpretation; or writing of the report. The corresponding author had full access to all the data in the study, and had final responsibility for the decision to submit for publication.

## Results

### Determinants and pathway to impact for CSR in sub-Saharan Africa

Among 19 countries with complete information in sub-Saharan Africa in the year 2011, economic development varied greatly (GDP/P range US$320–6500, median US$705, inter-quartile range [IQR] US$490–1000). CSRs varied from 150 in DR Congo to 2000 in Gambia (median 500, IQR 350–650). (Table 1) CSR was not linearly correlated with GDP/P (β = 0.07, 95% CI = -0.23 to 0.27).

**Table 1:** Detailed information on indicators in sub-Saharan Africa and in China

Region	Variable	Mean	Std. Error	Std. Deviation	Bootstrap ^a^		
					Std. Error	95% Confidence Interval	
						Lower	Upper
Sub-Saharan Africa	CSR	594.74	95.89	417.96	98.45	439.47	820.99
	GDP/P	1107.50	320.20	1395.72	318.90	645.85	1891.74
	Cataract surgeons per million	2.84	0.39	1.72	0.40	2.13	3.72
	General Doctors per 1000	0.11	0.02	0.10	0.02	0.07	0.16
	Ageing Proportion	2.93	0.07	0.31	0.07	2.81	3.08
	Male Proportion	49.67	0.14	0.62	0.14	49.40	49.92
	Income per capita	871.82	256.42	1117.72	255.55	504.19	1488.24
China	CSR	1125.42	121.64	677.25	121.73	894.17	1400.21
	GDP/P ^b^	8264.30	645.89	3596.18	624.73	7042.04	9537.51
	General Doctors per 1000	2.15	0.07	0.40	0.07	2.03	2.30
	Ageing Proportion	8.51	0.27	1.51	0.26	8.01	9.04
	Male Proportion	51.37	0.12	0.66	0.11	51.15	51.60
	Annual Consumption#	2857.40	232.03	1291.88	224.42	2435.56	3353.69
	Social Insurance scale#	162.90	19.24	107.15	19.14	129.32	207.11

^a^: 95% CI were based on 1000 bootstrap samples

^b^: converted into US dollars according to RMB: US dollars = 6.14: 1

**Fig. 1:**
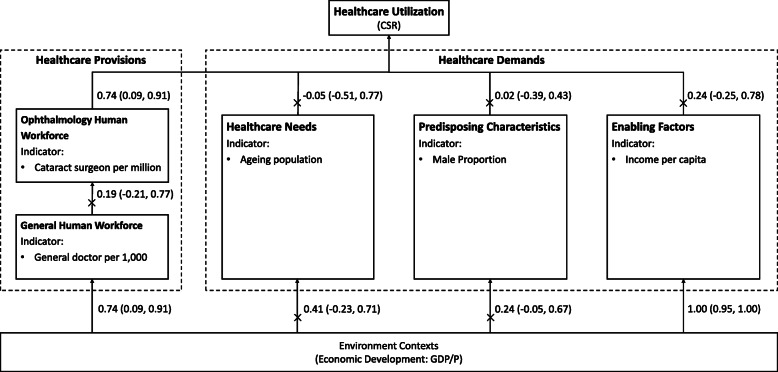
Andersen’s healthcare utilization model for Cataract Surgery Rate in sub-Saharan Africa. CSR, Cataract Surgery Rate; GDP/P, Gross Domestic Product per Capita; Numbers out of brackets stand for the standardized coefficients between the two ends of the wire; Numbers in brackets stand for the 95% confidence intervals of the standardized coefficients

After data standardization, CSR only showed a linear relationship in our model with the number of ophthalmologists per million population, which is by provision of healthcare service in Anderson’s terms. (Table 2) In addition, GDP/P was linearly correlated with the number of general practitioners per unit population, but not with the number of ophthalmologists. This suggests that increases in GDP/P may be associated with enhanced supply in the general medical workforce, but not in ophthalmology. The model for CSR in sub-Saharan Africa is summarized in Fig. 1.

**Table 2:** Standardized Pearson’s correlations among indicators in sub-Saharan Africa^a^

	CSR	GDP/P (US dollars)	Cataract surgeons per million	Doctors per 1000	Ageing Proportion	Male Proportion	Income per capita (US dollars)
CSR	1						
GDP/P	0.23(−0.25,0.78)	1					
Cataract surgeons per million	0.74*(0.09, 0.91)	0.29(− 0.12, 0.75)	1				
General Doctors per 1000	− 0.08(− 0.41, 0.52)	0.74*(0.30, 0.95)	0.19(− 0.21, 0.77)	1			
Ageing Proportion	−0.05(− 0.51, 0.77)	0.41(− 0.23, 0.71)	− 0.20(− 0.63, 0.40)	0.05(− 0.44, 0.53)	1		
Male Proportion	0.02(− 0.39, 0.43)	0.24(− 0.05, 0.67)	0.03(− 0.50, 0.48)	0.52*(0.22, 0.83)	0.04(− 0.39, 0.37)	1	
Income per capita	0.24(− 0.25, 0.78)	1.00*(0.95, 1.00)	0.31(− 0.10, 0.76)	0.72*(0.28, 0.94)	0.41(− 0.26, 0.71)	0.21(− 0.16, 0.64)	1

^a^: 95% CI were based on 1000 bootstrap samples

*: *P* < 0.05

### Determinants and pathway to impact for CSR in China

Among 31 administrative units in China in the year 2014, provincial economic development varied greatly (GDP/P range US$4300 to 17,100, median US$6600, IQR US$5700–10,300) [16], while CSRs ranged from 330 (Xinjiang Province) to 3807 (Shanghai) (median 976, IQR 642–1326). (Table 1). CSR was linearly correlated only with GDP/P (β = 0.12, 95% CI = 0.03 to 0.20).

Principal component analysis was applied to calculate the comprehensive score for ability to pay because the two raw indicators, health insurance investment per capita, and annual consumption per capita, were highly correlated (Pearson correlation = 0.87, 95%CI = 0.63 to 0.94). The result suggested the eigenvalue of the first component was 1.87, while the eigenvalue of the second component was only 0.14. Therefore, the first component was adequate to reflect represent ability to pay in our model.

**Fig. 2:**
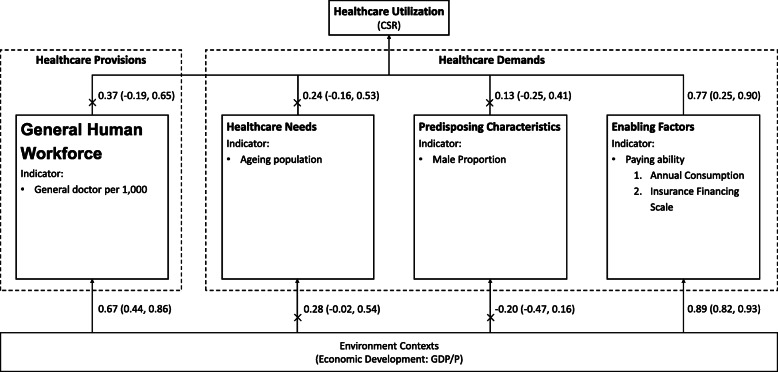
Andersen’s healthcare utilization model for Cataract Surgery Rate in China. CSR, Cataract Surgery Rate; GDP/P, Gross Domestic Product per Capita; Numbers out of brackets stand for the standardized coefficients between the two ends of the wire; Numbers in brackets stand for the 95% confidence intervals of the standardized coefficients

After data standardization, our model for determinants of CSR in China showed that ability to pay was the sole direct determinant of CSR. (Table 3) Therefore, CSR in China is principally determined by healthcare demand. The SEM analysis (Fig. 2) confirms that ability to pay is a mediator between GDP/P and CSR (*p* = 0.32, RMSEA = 0.07). This finding indicated that in China, economic development may be expected to lead to an increase in patients’ ability to pay, resulting in a rise in CSR.

**Table 3:** Standardized Pearson’s correlations among indicators in China^a^

	CSR	GDP/P	General Doctors	Ageing Proportion	Male Proportion	Ability to Pay
CSR	1					
GDP/P	0.63* (0.18, 0.79)	1				
General Doctors per 1000	0.37(−0.19, 0.65)	0.67*(0.44, 0.86)	1			
Ageing Proportion	0.24(−0.16, 0.53)	0.28(−0.02, 0.54)	0.09(− 0.19, 0.37)	1		
Male Proportion	0.13(−0.25, 0.41)	−0.20(− 0.47, 0.16)	−0.22(− 0.52, 0.06)	−0.26(− 0.62, 0.21)	1	
Ability to Pay	0.77*(0.22, 0.91)	0.89*(0.84, 0.95)	0.74*(0.45, 0.92)	0.25*(0.01, 0.48)	−0.11(− 0.39, 0.16)	1

^a^: 95% CI were based on 1000 bootstrap samples.*: *P* < 0.05

## Discussion

Our study creates models of CSR in China and sub-Saharan Africa, based on readily available data at population level and Andersen’s healthcare utilization model. Our principal finding is that the key determinants of CSR in these two settings are in fact very different. In sub-Saharan Africa, the primary determinant of cataract surgical delivery is in the service provision domain, specifically the supply of human resources (cataract surgeons.) The supply of ophthalmologists in this setting is not correlated with GDP/P, suggesting that economic development in Africa may not lead directly to an increase in supply of cataract- surgeons (or growth in CSR) without contributions from external factors. This lack of an association between GDP/P and CSR is in contradiction to what has been reported based on global statistics [5]. In addition, our result suggests that in China, CSR is determined primarily by demand side factors, specifically patients’ ability to pay, which is associated with GDP/P. This latter finding suggests that economic development in China may lead to further increases in CSR, as has been seen in recent years [5].

With regard to our findings for sub-Saharan Africa, infectious diseases and malnutrition are still severe in this region, and thus the elimination of infectious diseases and under-nourishment may be the prime missions of these healthcare systems [20]. Thus, increases in GDP/P might not drive the diagnosis and treatment of ophthalmological diseases, which are not usually lethal and potentially a lower priority [20].

Our findings regarding human resources as the major determinant of CSR in Africa is consistent with the existing literature indicating that a lack of ophthalmologists, especially surgeons, is the major barrier to the realization of VISION 2020 in this setting [21–23]. However, it should be noticed that a CSR of about 500 and with 2.9 cataract surgeons per million population means that each surgeon was performing less than 200 cataract operations per year, which was a very low workload. Therefore, the key issue was not the total number of ophthalmologist, but the equity of access of the ophthalmology healthcare service. In other words, we need to focus on the ophthalmology resources in the remote areas.

In addition, we found in the sub-Saharan Africa, increase in residents’ average income might not increase CSR. Two reasons should be noticed. First, there was few evidences suggesting that residents’ average income increase might strength spending power of the poorest sections of society in the sub-Saharan Africa, who typically were considered to dominate the untreated cataract blind population. Secondary, even if the poorest’ income increased, they did not necessarily spend the money on cataract surgery. Though some projects aimed at removing financial barriers (eg in the running of “free cataract camps”) may help patients access surgery, projects that remove financial barriers are quite different in impact for patients than income increase on a national level (GDP/P). “Free cataract camps” is a kind of subsidy specifically for cataract surgery, but income increase is not. For “free cataract camps”, cataract patients may have two options: either to receive free cataract surgery; or to give up the surgery without any other subsidies. In this case, the best choice for cataract patients may be free surgery. But the increase in income is not such an “All or none” issue. Imagine if we change the option of “free cataract camps” to: either accept free cataract surgery or give up surgery but receive financial assistance, what will happen? Perhaps many cataract patients will choose to receive subsidies for purchasing clean and nutritious food or treating other deadly diseases. Therefore, we believe that in very poor areas such as the sub-Saharan Africa, it is very important to improve the cataract patients’ ability to bear the surgery, but this improvement cannot be achieved by residents’ average income increase.

In China, our study highlights the importance of patients’ ability to pay as a determinant of CSR. Several researchers concur that an insufficient ability to pay has negative impacts on individual cataract surgery utilization [11]. In rural China, willingness to pay for cataract surgery was only US$55 ± 55 in 2007 [24], while 72.2% of those surveyed reported monthly incomes <US161 [25]. In urban areas, willingness to pay was approximately US$1000 in 2013. However, the real cost for monocular cataract surgery in urban has been reported much higher: US$1300 to 1600 in 2014 [26]. In summary, cataract surgery remains a large financial burden for many Chinese people.

In recent decades, many studies have been carried out in China to explore possible methods to improve the CSR. The existing literature indicates that the most effective ways to increase cataract surgical output in China is by lowering surgical fees [17–19], increasing health insurance reimbursement rates [16], and enhancing investment in outreach screening [15]. A study in rural northern China reported that cost was the most common barrier to cataract surgery [17], while in rural Shaanxi province, provision of free cataract surgery was more effective in increasing uptake of cataract surgery informational reminders, reimbursement of transportation fees, or the offer of free rides [18]. Another study in rural Guangdong found that the impact of a reduction in surgical fees on surgical uptake was approximately twice that of training local surgeons [19]. Finally, a study in Chongqing found that an increase in access to health insurance significantly improved utilization of cataract surgery, especially in rural areas [16].

Limitations to the present study must be acknowledged. The sample size is necessarily limited to a modest number of African countries and Chinese administrative units for whom the full set of data necessary for our model is publicly available. Hence, we had not sufficient evidence to believe that the data used in this study meets the multivariate normality assumption. Therefore, the traditional parameter method is not suitable, and the Bollen-Stiner Bootstrap structural equation is a better choice [27]. Further studies with larger samples are needed to describe the relationships between CSR and its impact factors in greater detail. Another limitation is the choice of economic indicator. In our study, GDP per capita was used to reflect the economic development level. However, in addition to GDP, GNI is another commonly used indicator. Although the difference between these two indictors is not obvious, GNI may be more appropriate for the analysis of Africa.

## Conclusions

The study creates Andersen’s healthcare utilization framework with Bollen-Stiner Bootstrap structural equation modeling, which can be a powerful and feasible method for fast evaluation of health service at population level, to clarify the relationships among potential determinants of CSR in very different settings, and points out possible pathways. In sub-Saharan Africa, CSR is determined by health care provision. Local economic development may not directly influence CSR because many other health problems need to be solved more urgently; therefore, in order to increase CSR, international assistance is crucial, and the form should be the project that directly provides free cataract surgery, such as “free cataract camps”. In China, CSR is determined principally by residents’ ability to pay, which can be enhanced with economic development, such as strengthening social insurance, and reducing surgical fees.

## Supplementary Information

**Additional file 1: Supplement 1.** Andersen’s healthcare utilization model for Cataract Surgery Rate

## Data Availability

The datasets used and/or analysed during the current study are available from the corresponding author on reasonable request.

## Abbreviations

CSR: Cataract surgical rate
GDP/P: Gross domestic product per capita
RMSEA: Root Mean Square Error of Approximation
CI: Confidence Intervals
WHO: World Health Organization
LMICs: Low and middle income countries
SDGs: Sustainable Development Goals
HSR: Health service research
SEM: Structural equation modeling
US: United States
IQR: Interquartile range
GNI: Gross national income
