# Supplementary material for: Andersen’s utilization model for cataract surgical rate and empirical evidence from economically-developing areas
Source: BMC Ophthalmol. 2021 Feb 26;21:107. doi: 10.1186/s12886-021-01858-x (PMC7908707; doi:10.1186/s12886-021-01858-x)
Supplement: Supplementary file 1 — Additional file 1: Supplement 1. Andersen’s healthcare utilization model for Cataract Surgery Rate [file 12886_2021_1858_MOESM1_ESM.pptx]

## Slide 1
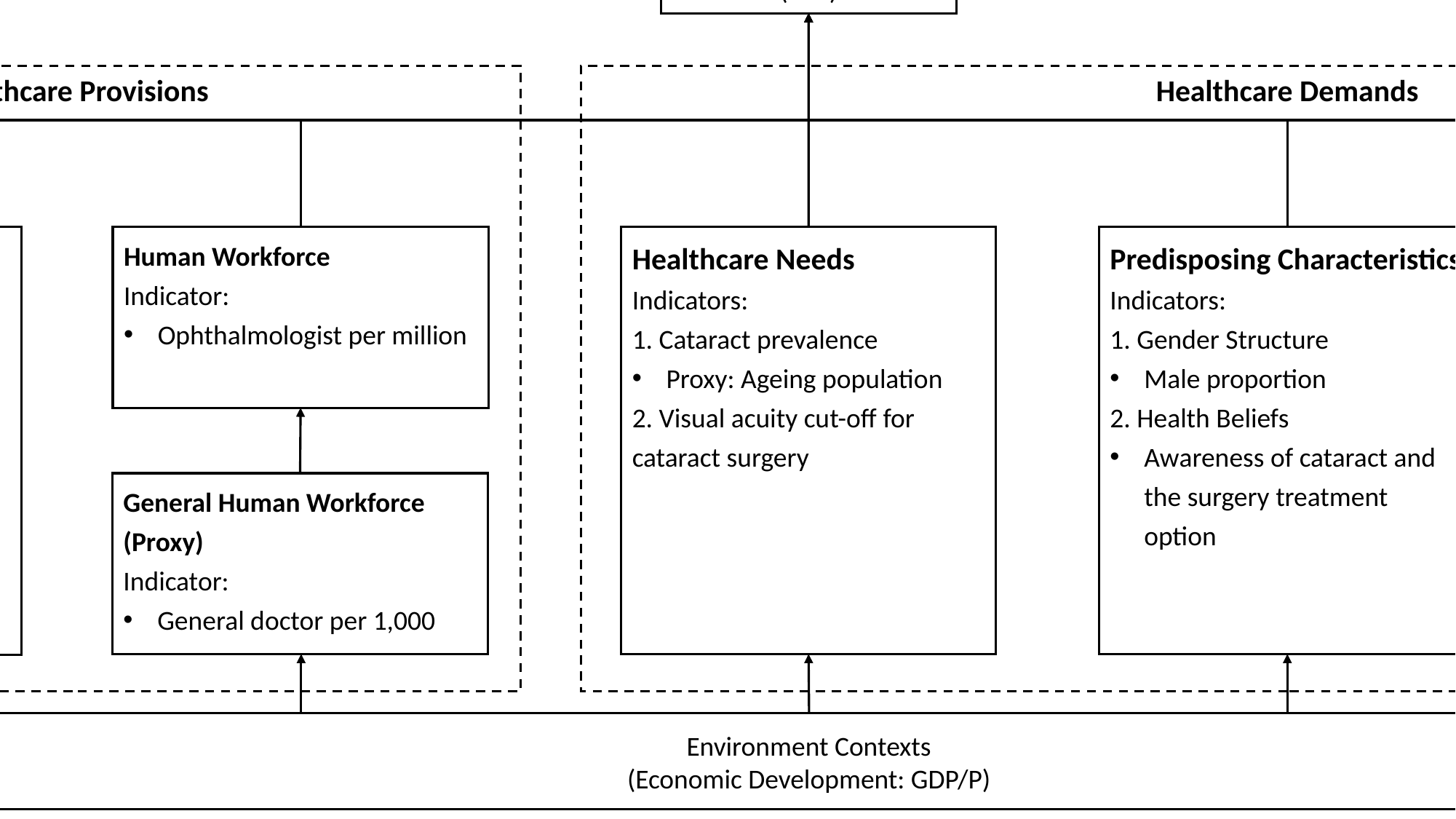

Healthcare Utilization
(CSR)
Healthcare Provisions
Healthcare Demands
Predisposing Characteristics
Indicators:
1. Gender Structure
Male proportion
2. Health Beliefs
Awareness of cataract and the surgery treatment option
Enabling Factors
Indicators:
1. Paying Ability
Social insurance financing scale per capita
Average annual consumption/income scale
Equipment
Indicator:
Ophthalmic equipment resources
Human Workforce
Indicator:
Ophthalmologist per million
Healthcare Needs
Indicators:
1. Cataract prevalence
Proxy: Ageing population
2. Visual acuity cut-off for cataract surgery
General Human Workforce
(Proxy)
Indicator:
General doctor per 1,000
Environment Contexts
(Economic Development: GDP/P)
CSR: Cataract Surgery Rate; GDP/P: Gross Domestic Product per Capita
Supplement 1. Andersen’s healthcare utilization model for Cataract Surgery Rate
